# Study on the Role and Mechanism of γδ T Cells in Atherosclerosis Under a High-Fat Diet

**DOI:** 10.31083/RCM48002

**Published:** 2026-05-11

**Authors:** Qinning Zhang, Xiaoxu Zhang, Meng Cao, Junbai Ma, Ru Yan, Hao Wang, Shaobin Jia

**Affiliations:** ^1^The First Clinical College of Ningxia Medical University, General Hospital of Ningxia Medical University, 750001 Yinchuan, Ningxia, China; ^2^Department of Gastroenterology, General Hospital of Ningxia Medical University, 750001 Yinchuan, Ningxia, China; ^3^Department of Clinical Nursing, School of Nursing, Ningxia Medical University, 750001 Yinchuan, Ningxia, China; ^4^Department of Pathogenic Microbiology and Immunology, Institute of Basic Medical College, Ningxia Medical University, 750001 Yinchuan, Ningxia, China; ^5^School of Laboratory Medicine, Ningxia Medical University, 750001 Yinchuan, Ningxia, China; ^6^Department of Cardiovascular Medicine, General Hospital of Ningxia Medical University, 750001 Yinchuan, Ningxia, China

**Keywords:** γδ T cells, atherosclerosis, hyperlipidemia, inflammatory cytokines, gut microbiota

## Abstract

**Background:**

This study aimed to investigate the effects of γδ T cell inhibition under a high-fat diet (HFD) on metabolic function, immune inflammation, gut microbiota, and atherosclerosis (AS) progression in *ApoE*^-/-^ mice.

**Methods:**

*ApoE*^-/-^ mice were assigned to three groups: a control group (normal diet), a model group (HFD), and an intervention group (HFD + γδ T cell receptor (TCR) monoclonal antibody). After 12 weeks, flow cytometry was used to assess γδ T cell levels, and cytokines (interferon-gamma (IFN-γ), IL-17A) were measured. Inflammatory markers in blood and adipose tissue were quantified, gut microbiota composition was analyzed via fecal metagenomics, and atherosclerosis was evaluated using Oil Red O, Masson's trichrome, and hematoxylin and eosin (HE) staining methods.

**Results:**

The HFD activated γδ T cells and increased pro-inflammatory cytokines in *ApoE*^-/-^ mice. Treatment with the γδ TCR monoclonal antibody suppressed γδ T cells, reduced IFN-γ and IL-17A expression, improved lipid profiles, and decreased tumor necrosis factor-alpha (TNF-α), IL-1β, and IL-6 levels. Gut microbiota analysis showed an increase in beneficial bacteria, and histological staining (Oil Red O, HE, and Masson's trichrome) confirmed a reduction in atherosclerotic lesion burden.

**Conclusion:**

The γδ T cells contribute to AS development under the HFD. Inhibition of γδ T cells reduces inflammation, improves gut microbiota composition, and attenuates atherosclerosis progression.

## 1. Introduction

Atherosclerosis (AS) is a critical pathological basis for ischemic heart disease and stroke. Its characteristic lesion involves the slow accumulation and complex transformation of lipids, immune cells, foam cells, smooth muscle cells, and necrotic cell debris within the intimal space beneath the monolayer of vascular endothelial cells (ECs) [1]. Atherosclerosis has become a major research focus in the field of cardiovascular diseases [2]. In recent years, extensive exploration of preclinical models and emerging evidence from human clinical studies have established the pivotal role of the immune system in driving the initiation and progression of atherosclerotic lesions. Among the immune components, the T-cell family serves as a key driver and regulator across all stages of AS development [3].

T cells are named after their development in the thymus and are characterized by numerous important membrane molecules on their surface, which play key roles in antigen recognition, activation, proliferation, differentiation, and effector functions. The T cell receptor (TCR), a specific receptor on the T cell surface that recognizes and binds to antigens, is a hallmark of all T cells [4].

The roles of different T cell subsets in AS have been extensively studied [5]. CD4+ T cell subsets can influence AS through immune activation or suppression, or by assisting B cells in antibody production, resulting in diverse effects on the disease. Among these, Th1 cells are the most prominent CD4+ T cell subtype in atherosclerotic plaques, contributing to inflammation by secreting cytokines such as interferon-gamma (IFN-γ) and tumor necrosis factor-alpha (TNF-α) [6].

The immunoregulatory relationship between T cells and the gut microbiota has become a major research focus. Current perspectives suggest a “mutualistic” interaction, where the plasticity of each component modulates the other. Symbiotic microbiota regulate the development, differentiation, and function of T cells through various mechanisms, including metabolic products, antigen presentation, intestinal barrier permeability, and immune tolerance. Conversely, T cells, through the coordinated activity of different subsets, regulate the balance and diversity of gut microbial communities [7]. Although γδ T cells comprise only a small fraction of circulating T cells (approximately 1–5%), they are highly enriched in the intestinal immune system. Studies have indicated that changes in the gut microbiota can influence cytokine secretion and epigenetic characteristics of intraepithelial γδ T cells in the intestinal mucosa [8, 9].

The pathogenesis of atherosclerosis is complex, involving interactions between metabolic abnormalities and immune responses that result in chronic inflammation of the arterial wall. As a subset of unconventional T cells, γδ T cells play a critical role in maintaining immune homeostasis. This study aims to explore the role and mechanisms of γδ T cells in atherosclerosis, providing novel insights and strategies for its prevention and treatment.

## 2. Materials and Methods

### 2.1 Ethical Approval, Experimental Animals, Housing and Husbandry

This study was approved by the Animal Welfare Ethics Committee of Ningxia Medical University (Ethics Approval No. IACUC-NYLAC-2023-164). A total of 42 male *ApoE*^-⁣/-^ mice (6–8 weeks old) were purchased from Beijing Charles River Laboratory Animal Technology Co., Ltd. (Beijing, China). Mice were housed at the Experimental Animal Center of Ningxia Medical University under specific pathogen-free (SPF) conditions at 22 ± 2 °C, 50%–60% relative humidity, and a 12-h light/dark cycle, with ad libitum access to food and water. Mice were group-housed (6 mice per cage) with standard bedding and environmental enrichment (nesting material).

Animals were monitored twice weekly for general health status (activity, grooming, posture, food/water intake) and body weight. Humane endpoints were predefined as persistent severe lethargy, inability to access food/water, or >20% body-weight loss, at which point animals would be euthanized.

For terminal procedures, anesthesia was induced with 3% isoflurane and maintained at 1.5% isoflurane (I8000, Solarbio, Beijing, China). Euthanasia by cervical dislocation was performed only after loss of righting and corneal reflexes. Death was confirmed by cessation of respiration and heartbeat. All operators were professionally trained. Isoflurane inhalation anesthesia was chosen due to rapid onset/offset and to avoid injection-related distress, consistent with animal welfare principles.

### 2.2 Study Design, Experimental Unit, Sample Size Rationale, and Grouping 

All male mice underwent a two-week acclimation period on a standard diet prior to the experiment to stabilize baseline conditions, as male mice were selected to minimize the potential confounding effects of sex hormone-related metabolic variability.

Experimental unit. The experimental unit was the individual mouse. To minimize potential cage effects, cage allocation and cage position were balanced across groups as feasible.

Study structure. The study consisted of two parts: (i) an antibody validation experiment and (ii) a modeling intervention experiment.

Sample size rationale and animal flow. The total sample size was based on feasibility and prior similar *ApoE*^-⁣/-^ high-fat diet studies; no formal a priori power calculation was performed (exploratory design). Six mice were used for antibody validation (2 groups, n = 3 per group), and the remaining 36 mice were used for the 12-week modeling intervention experiment (3 groups, n = 12 per group). The number of animals included in each downstream analysis is reported in the corresponding figure legends; any exclusions are described under the predefined criteria (Section 2.2.1) and reported in the Results. In addition, fecal samples were collected from all mice for gut microbiota assessment. However, due to cost considerations, fecal samples from 6 mice per group were randomly selected for shotgun metagenomic sequencing (n = 6 per group). The sequencing subset was selected using a random number table by an investigator not involved in downstream outcome assessment, and the corresponding sample sizes are indicated in the relevant figure legends.

Randomisation and allocation. In the antibody validation experiment, six mice were randomly selected and divided into two groups: a 0.9% NaCl control group and a UC7-13D5 injection group. In the modeling intervention experiment, the remaining 36 mice were randomly allocated to three groups using a random number table to minimize selection bias: normal chow diet group (NCD), high-fat diet group (HFD), and high-fat diet+antibody group (HFD+Antibody). Randomisation was performed by an investigator not involved in outcome assessment; group codes were retained until completion of blinded analyses. Baseline body weight was checked to ensure comparable distribution across groups.

Diets. Each modeling cycle lasted 10 days, with intraperitoneal injections administered for 3 consecutive days followed by a 7-day interval. This cycle was repeated over a total 12-week intervention period. The NCD group received a normal diet (MD17121, Jiangsu Medison Biopharmaceutical Co., Ltd., Yangzhou, Jiangsu, China), while the HFD and HFD+Antibody groups received a high-fat diet (40% fat Kcal %, 1.25% cholesterol; MD12017, Jiangsu Medison Biopharmaceutical Co., Ltd., Yangzhou, Jiangsu, China).

Handling/injection control. To control for handling and injection-related stress, mice in the NCD and HFD groups received intraperitoneal injections of 0.9% NaCl (Kelun Pharmaceutical Co., Ltd., Chengdu, Sichuan, China) on the same schedule as the antibody group.

#### 2.2.1 Predefined Inclusion and Exclusion Criteria

Inclusion criteria were: healthy male *ApoE*^-⁣/-^ mice aged 6–8 weeks after acclimation with body weight within the supplier’s reference range and no overt signs of illness. Prespecified exclusion criteria included: (i) accidental death unrelated to the intervention, (ii) severe illness requiring humane euthanasia, (iii) failed intraperitoneal injection (e.g., leakage), and (iv) fecal metagenomic samples failing quality-control thresholds. Exclusions were documented prior to unblinding and are reported in the Results.

#### 2.2.2 Blinding

Outcome assessments for histology quantification and ImageJ measurements were performed blinded to group allocation. Investigators conducting statistical analyses were blinded to group codes until analyses were finalized. For metagenomic sequencing, fecal samples were labeled using anonymized codes prior to shipment and analysis. Personnel administering injections were not blinded due to the nature of the intervention.

### 2.3 Sample Collection and Processing

Fecal samples were collected in the morning on non-injection days and immediately stored at –80 °C for subsequent gut microbiota analysis. At the end of the intervention period, mice were anesthetized with isoflurane. Blood was collected via orbital sinus into EDTA-coated tubes (367841, BD Biosciences, Franklin Lakes, NJ, USA) as a terminal procedure by trained personnel, and hemostasis was ensured. Following blood collection, 0.9% NaCl was used for cardiac perfusion, and the heart, aorta, intestine, and inguinal adipose tissues were harvested. Portions of tissues were fixed in 4% paraformaldehyde (P1110, Solarbio, Beijing, China) for histological analyses.

### 2.4 Experimental Methods

Intraperitoneal injections were administered in the lower right abdomen with a total volume of 0.1 mL per injection. Mice received either 0.9% NaCl or 200 µg UC7-13D5 antibody (Ultra-LEAF™ Purified anti-mouse TCR γ/δ Antibody, 107519, BioLegend, San Diego, CA, USA). The needle was inserted into the peritoneal cavity and the solution was injected slowly to reduce discomfort.

Antibody validation experiment. Mice received intraperitoneal injections for 3 consecutive days and were sacrificed 7 days later to assess the inhibitory effect of UC7-13D5 on γδ T cells.

Flow cytometry. Flow cytometry (CytoFLEX V2-B4-R2, Beckman Coulter, Brea, CA, USA) was used to quantify γδ T-cell frequency and intracellular IFN-γ and IL-17A expression in peripheral blood mononuclear cells (PBMCs). The antibodies used for flow cytometry were: CD3e Monoclonal Antibody PerCP-Cyanine5.5, TCR gamma/delta Monoclonal Antibody FITC, IFN gamma Monoclonal Antibody APC, IL-17A Monoclonal Antibody PE. All four antibodies were purchased from Thermo Fisher Scientific (Waltham, MA, USA).

Lipid profiling. Triglycerides (TG), total cholesterol (TC), high-density lipoprotein cholesterol (HDL-C), and low-density lipoprotein cholesterol (LDL-C) were measured using an automated biochemical analyzer (Chemray 800, Rayto Life and Analytical Sciences Co., Ltd., Shenzhen, Guangdong, China).

Plasma cytokines. Plasma TNF-α, IL-1β, and IL-6 were measured by a third-party laboratory using multiplex flow cytometry (ABplex-100, ABclonal Technology Co., Ltd., Wuhan, Hubei, China).

qRT-PCR. Total RNA was extracted from adipose tissue using TRIzol reagent (Invitrogen, Carlsbad, CA, USA). RNA concentration and purity were assessed using a NanoDrop spectrophotometer (Thermo Fisher Scientific, Waltham, MA, USA). cDNA was synthesized from 1 µg RNA using PrimeScript RT Reagent Kit (TaKaRa Bio, Kyoto, Kansai region, Japan). qPCR was performed using SYBR Premix Ex Taq II (TaKaRa Bio, Kyoto, Kansai region, Japan). Relative expression was calculated using the 2^-Δ⁢Δ⁢Ct^ method.

### 2.5 Histological Analysis

Histological analysis included Oil Red O staining, hematoxylin and eosin (HE) staining, and Masson trichrome staining, which were used to assess lipid deposition, cellular structure, and fibrosis, respectively. Oil Red O staining: Performed on the aortic surface and aortic sinus for whole-field staining, allowing visualization of lipid deposition under a microscope to quantify the lipid burden associated with atherosclerosis. HE staining: Applied to 6-µm-thick frozen sections of the aortic sinus. After fixation and staining, the sections were examined under a microscope to observe cellular structures, tissue morphology. Masson trichrome staining: Conducted on aortic sections to stain collagen fibers, enabling quantitative analysis of fibrous tissue deposition in atherosclerotic lesions. This was used to evaluate the progression of fibrosis in the diseased tissues.

Image analysis was performed using ImageJ version 1.8.0 (National Institutes of Health, Bethesda, MD, USA). The lesion area index was defined as the proportion of the aortic lumen area occupied by atherosclerotic lesions. All analysts involved in histological quantification were blinded to group allocation.

### 2.6 Gut Microbiota Analysis

Fecal metagenomic sequencing was conducted by Shanghai Majorbio Bio-Pharm Technology Co., Ltd. (Shanghai, China). DNA was extracted from fecal samples and paired-end libraries were constructed using the NEXTFLEX™ Rapid DNA-Seq Kit (PerkinElmer, Waltham, MA, USA), followed by high-throughput sequencing using Illumina NovaSeq Reagent Kits (Illumina, San Diego, CA, USA). Bioinformatics analyses were performed on the company’s online platform, including quality control, taxonomic and functional annotation, and community profiling.

Alpha diversity was calculated from fecal metagenomic relative abundance profiles. Overall differences among groups were tested using the Kruskal-Wallis test, followed by Dunn’s post hoc comparisons with Benjamini-Hochberg FDR correction. Beta diversity was assessed using Bray-Curtis dissimilarities and visualized by principal coordinates analysis (PCoA) and non-metric multidimensional scaling (NMDS), with between-group differences tested by permutational multivariate analysis of variance (PERMANOVA). Differential abundance was evaluated using the Wilcoxon rank-sum test with FDR correction, and further characterized by LEfSe (α = 0.05; LDA score >2).

In evaluating the impact of γδ T-cell inhibition on the gut microbiota under high-fat diet conditions, we only compared the HF and HF+Ab groups. This decision was made because we considered that including the NCD group in the core comparison could introduce unnecessary confounding factors.

### 2.7 Outcomes and Statistical Analysis

Outcome measures. The primary outcome was atherosclerotic lesion burden (lesion area index). Secondary outcomes included circulating lipid parameters, plasma inflammatory cytokines, γδ T-cell frequency/cytokine expression, and gut microbiota composition and diversity.

Statistical analyses were performed using GraphPad Prism 10.0 (GraphPad Software, San Diego, CA, USA) and SPSS 28.0 (IBM Corp., Armonk, NY, USA). Normality was assessed using the Shapiro-Wilk test and homogeneity of variance using Levene’s test. All tests were two-sided with α = 0.05. Normally distributed data are presented as mean ± SD. For two-group comparisons, Student’s *t*-test was used for normal data and Mann-Whitney U test for non-normal data. For multiple-group comparisons at a single time point, one-way ANOVA followed by Dunnett’s post hoc test was applied. For longitudinal measures (body weight and food intake), repeated-measures ANOVA was used; sphericity was assessed with Mauchly’s test and Greenhouse-Geisser correction was applied when violated. No data points were excluded unless prespecified exclusion criteria were met (Section 2.2.1). Investigators involved in statistical analyses were blinded to group allocation as described above.

## 3. Results

**Fig. 1.**
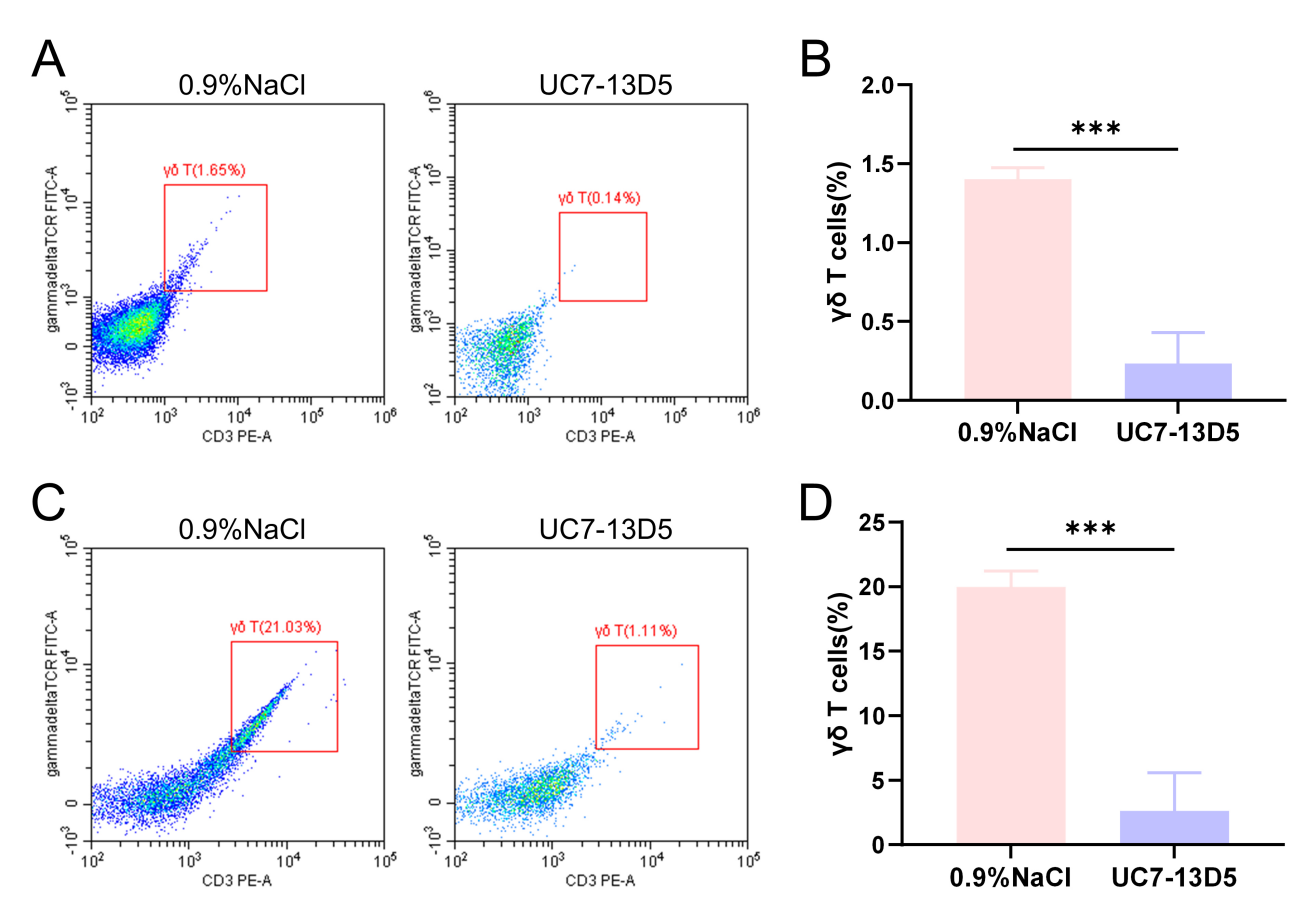
**Validation results of the UC7-13D5 antibody experiment**. (A,B) Flow cytometry results and statistical analysis of PBMCs from the control and antibody-treated groups. (C,D) Flow cytometry results and statistical analysis of IELs from the control and antibody-treated groups. Compared with the control group, ***p < 0.001. Sample size: n = 3 per group. Statistics: Data with a normal distribution were analyzed using Student’s *t*-test (two groups) or one-way ANOVA (multiple groups). Non-normally distributed data were analyzed using rank-based tests, including the Mann-Whitney U test (two groups) or the Kruskal-Wallis test (multiple groups). PBMCs, peripheral blood mononuclear cells; IELs, intraepithelial lymphocytes.

**Fig. 2.**
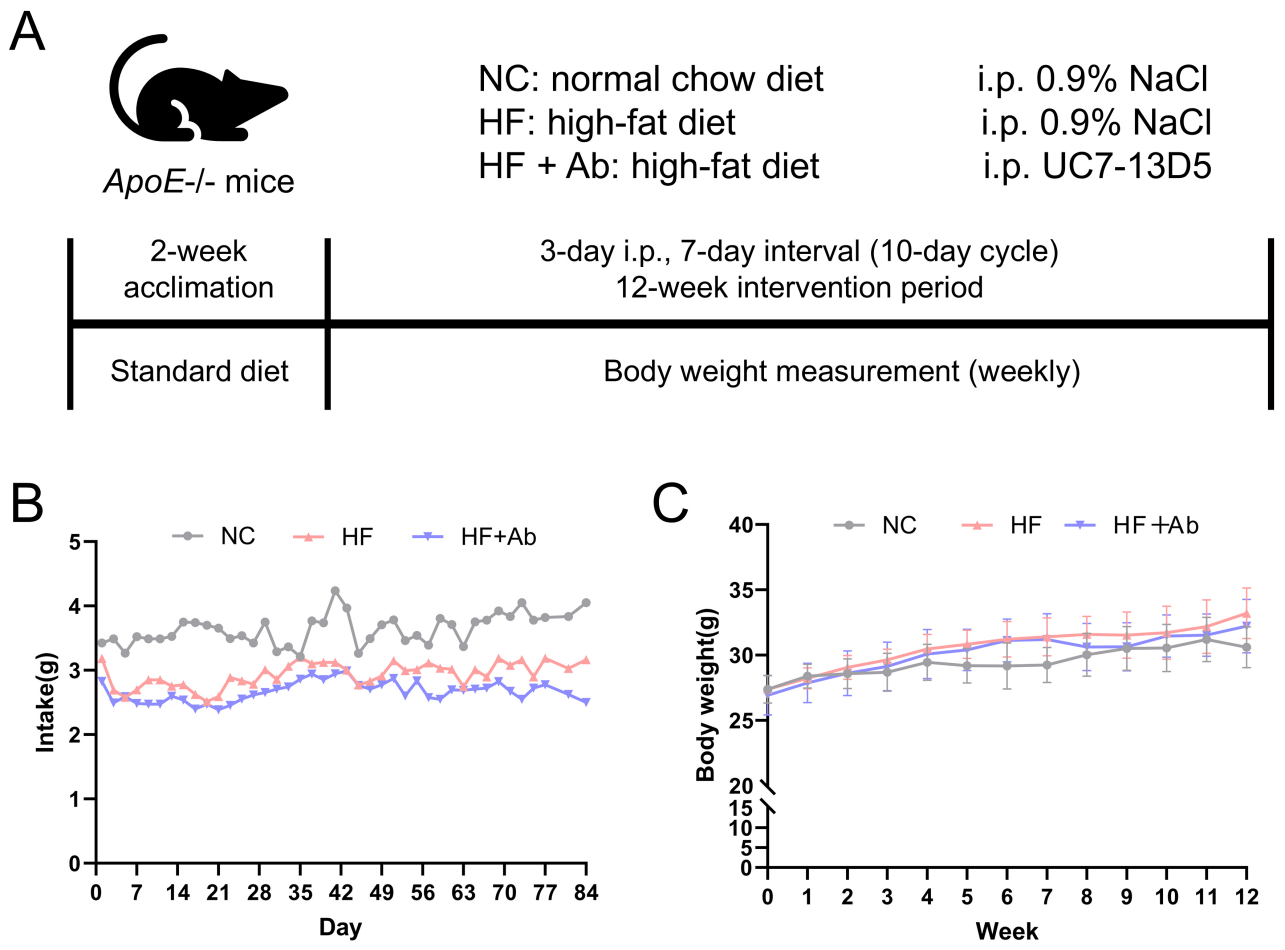
**Overview of the intervention workflow and food intake/body weight monitoring throughout the experiment**. (A) Schematic timeline of the experimental design. (B) Food intake was measured every 3 days and expressed as g/day/mouse. (C) Body weight was measured weekly. Data are presented as mean ± SD. Sample size: n = 12 per group. Statistics: Longitudinal data were analyzed by repeated-measures ANOVA (SPSS). Mauchly’s test indicated violation of sphericity (*p* < 0.05); therefore, Greenhouse–Geisser correction was applied. For food intake, neither the main effect of time (F = 4.008, *p* = 0.070) nor the time × group interaction was significant (F = 1.416, *p* = 0.328). For body weight, the main effect of time was significant (F = 93.050, *p* < 0.001), whereas the time × group interaction was not significant (F = 0.722, *p* = 0.542).

**Fig. 3.**
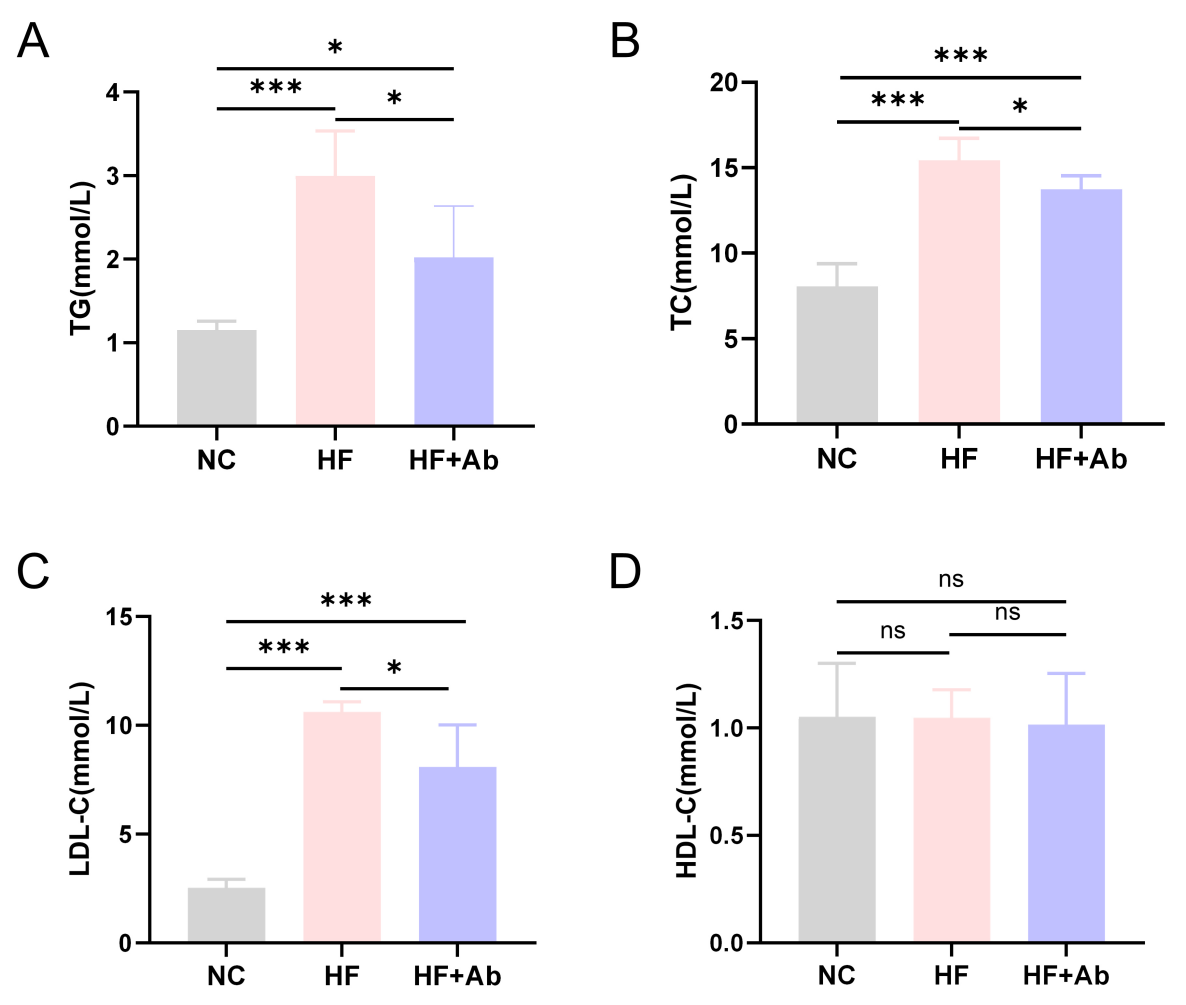
**Lipid analysis results of mice in each group during the intervention experiment**. (A) Triglycerides (TG). (B) Total cholesterol (TC). (C) Low-density lipoprotein cholesterol (LDL-C). (D) High-density lipoprotein cholesterol (HDL-C). Comparison among groups: *p < 0.05, ***p < 0.001, ns: no significant difference. Sample size: n = 12 per group. Statistics: Data with a normal distribution were analyzed using Student’s *t*-test (two groups) or one-way ANOVA (multiple groups). Non-normally distributed data were analyzed using rank-based tests, including the Mann-Whitney U test (two groups) or the Kruskal-Wallis test (multiple groups).

**Fig. 4.**
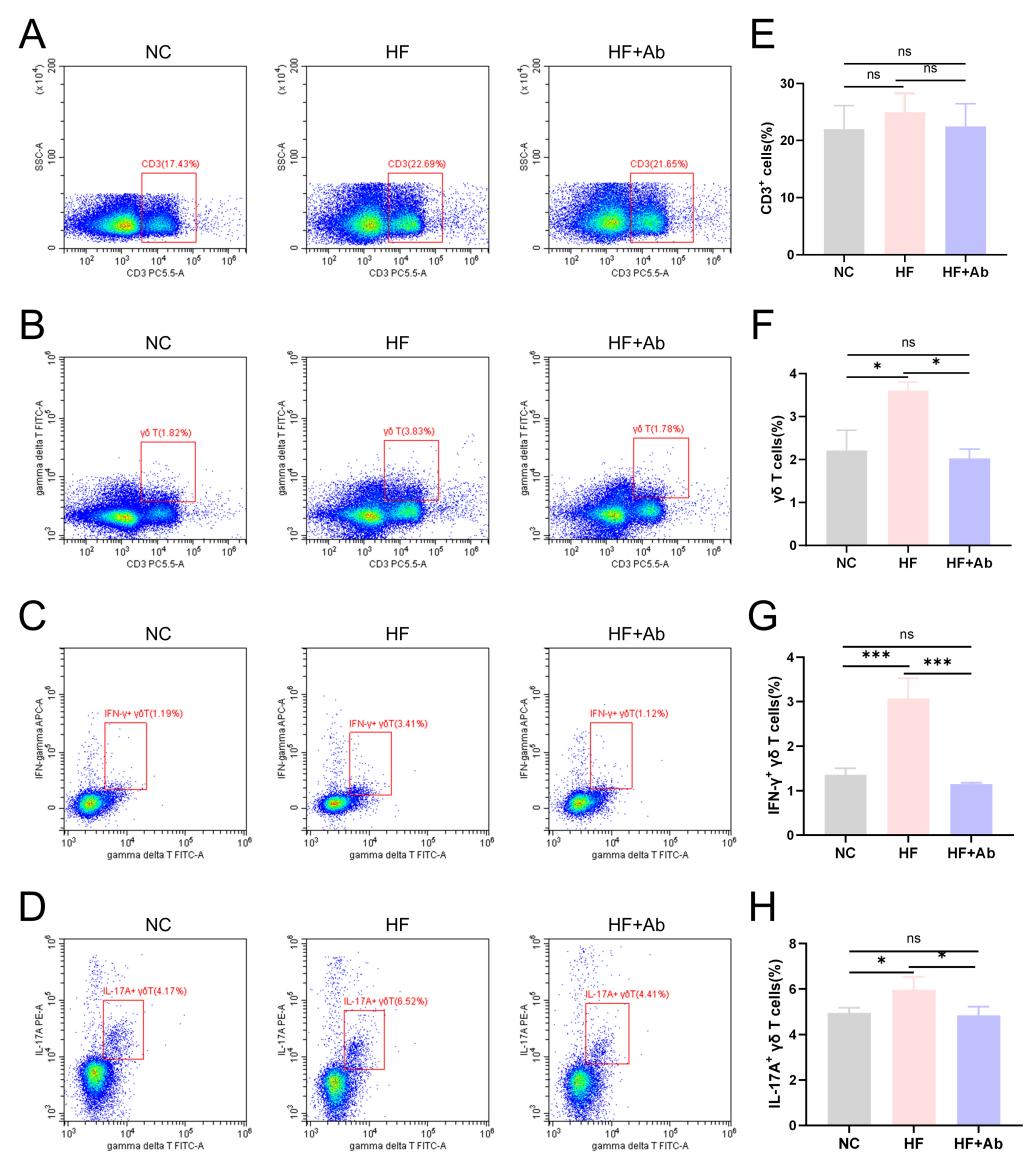
**Flow cytometry results of γδ T cells and their cytokines in PBMCs of mice from each group in the intervention experiment**. (A,E) Flow cytometry plots and statistical results of CD3+ T cells. (B,F) Flow cytometry plots and statistical results of CD3+ γδT+ cells. (C,G) Flow cytometry plots and statistical results of IFN-γ+ γδ T cells. (D,H) Flow cytometry plots and statistical results of IL-17A+ γδ T cells. Group comparisons: *p < 0.05, ***p < 0.001, ns: no significant difference. Sample size: n = 12 per group. Statistics: Data with a normal distribution were analyzed using Student’s *t*-test (two groups) or one-way ANOVA (multiple groups). Non-normally distributed data were analyzed using rank-based tests, including the Mann-Whitney U test (two groups) or the Kruskal-Wallis test (multiple groups). IFN-γ, interferon-gamma.

**Fig. 5.**
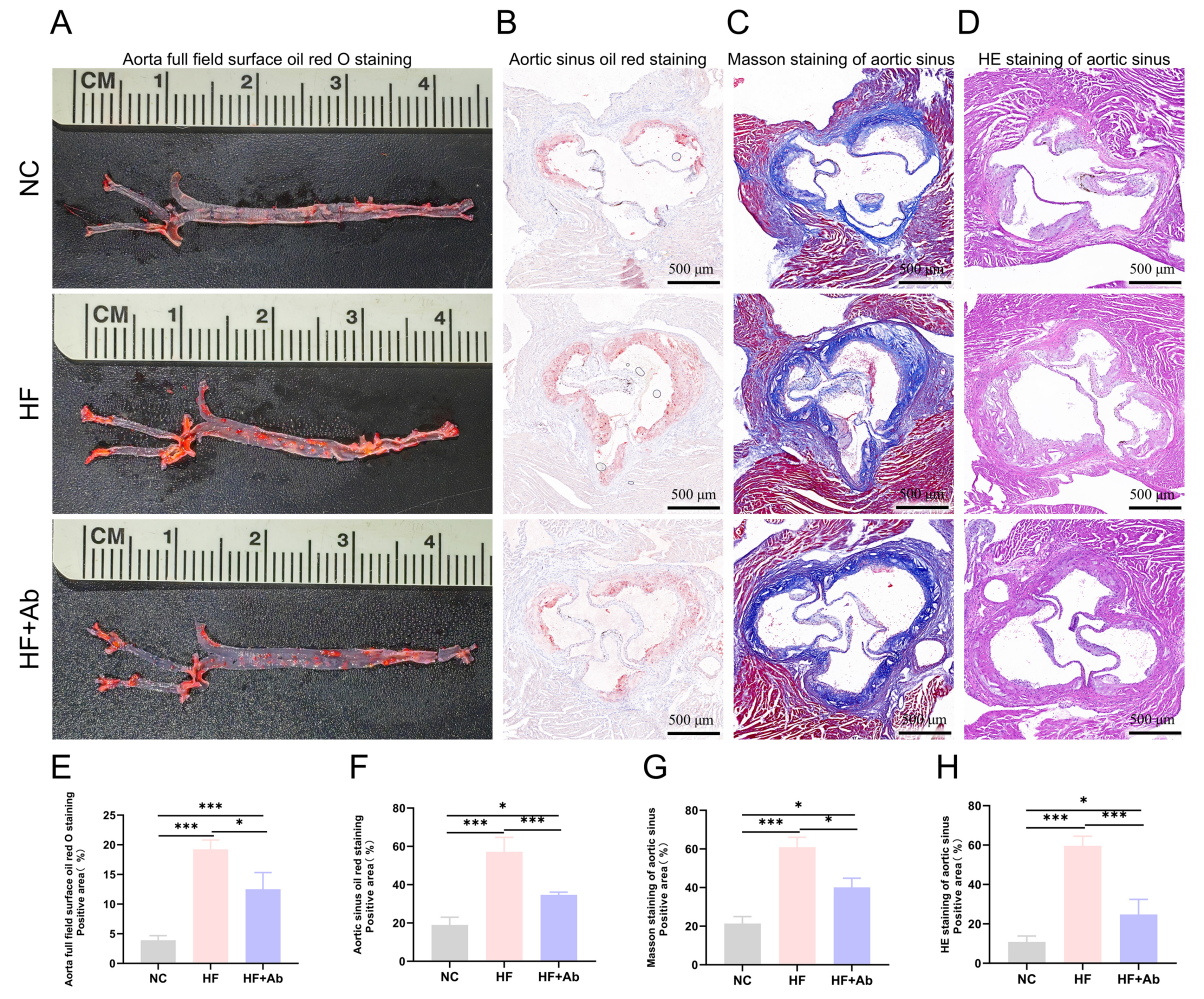
**Histological staining and analysis of atherosclerosis in the aorta of mice from each group in the intervention experiment**. (A,E) Oil Red O staining and statistical analysis of positive area on the aortic surface. (B,F) Oil Red O staining and statistical analysis of positive area in the aortic sinus. (C,G) Masson staining and statistical analysis of positive area in the aortic sinus. (D,H) HE staining and statistical analysis of necrotic core area in the aortic sinus. Group comparisons: *p < 0.05, ***p < 0.001. Sample size: n = 12 per group. Statistics: Data with a normal distribution were analyzed using Student’s *t*-test (two groups) or one-way ANOVA (multiple groups). Non-normally distributed data were analyzed using rank-based tests, including the Mann-Whitney U test (two groups) or the Kruskal-Wallis test (multiple groups). Scale bar = 500 µm.

**Fig. 6.**
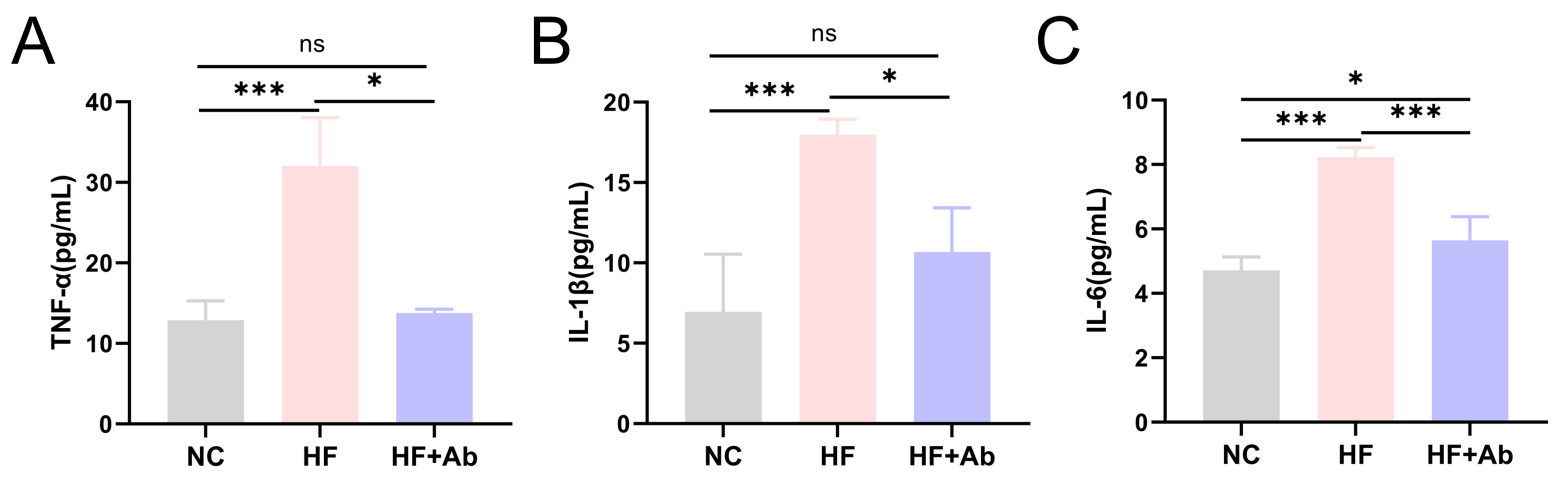
**Inflammatory cytokine levels in peripheral blood of mice from each group in the intervention experiment**. (A) TNF-α. (B) IL-1β. (C) IL-6. Group comparisons: *p < 0.05, ***p < 0.001, ns: no significant difference. Sample size: n = 12 per group. Statistics: Data with a normal distribution were analyzed using Student’s *t*-test (two groups) or one-way ANOVA (multiple groups). Non-normally distributed data were analyzed using rank-based tests, including the Mann-Whitney U test (two groups) or the Kruskal-Wallis test (multiple groups). TNF-α, tumor necrosis factor-alpha.

**Fig. 7.**
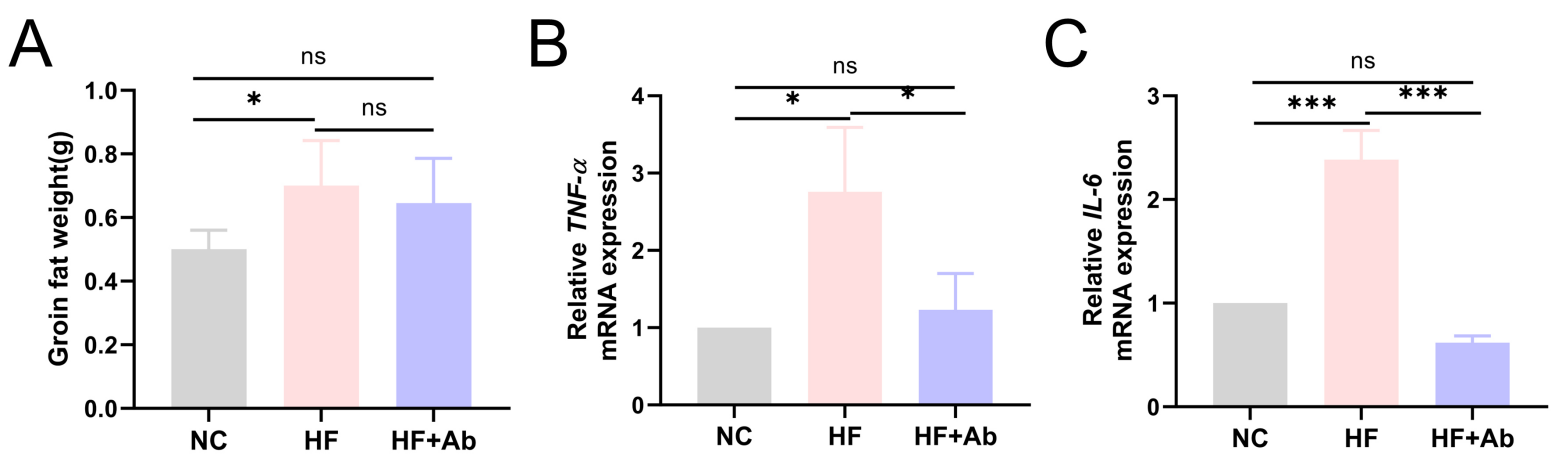
**Quantitative analysis and mRNA expression of inflammatory cytokines in inguinal adipose tissue of mice from each group in the intervention experiment**. (A) Groin fat weight. (B) *TNF-α*. (C) *IL-6*. Group comparisons: *p < 0.05, ***p < 0.001, ns: no significant difference. Sample size: n = 12 per group. Statistics: Data with a normal distribution were analyzed using Student’s *t*-test (two groups) or one-way ANOVA (multiple groups). Non-normally distributed data were analyzed using rank-based tests, including the Mann-Whitney U test (two groups) or the Kruskal-Wallis test (multiple groups).

**Fig. 8.**
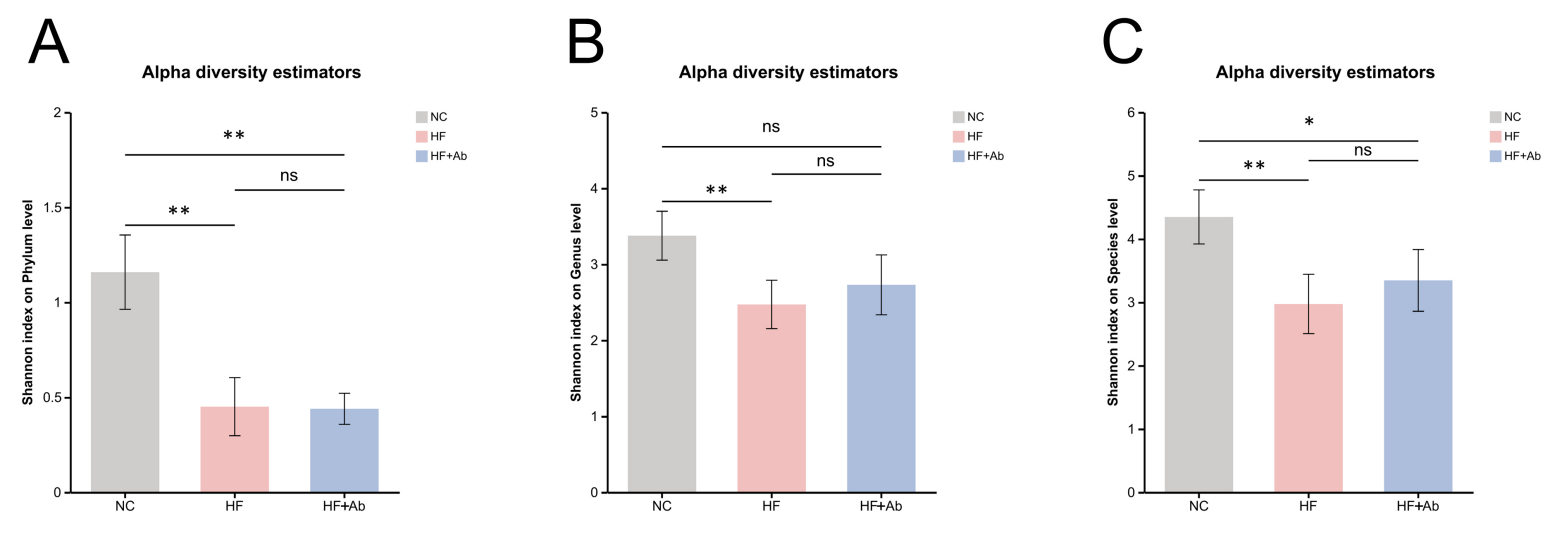
**Alpha-diversity analysis of fecal metagenomic sequencing across groups**. Shannon index comparisons at the phylum (A), genus (B), and species (C) levels. Comparison among groups: *p < 0.05, **p < 0.01, ns: no significant difference. Sample size: n = 6 per group. Statistics: Overall group differences were assessed using the Kruskal-Wallis test, followed by Dunn’s post hoc test with Benjamini-Hochberg FDR correction for multiple comparisons.

**Fig. 9.**
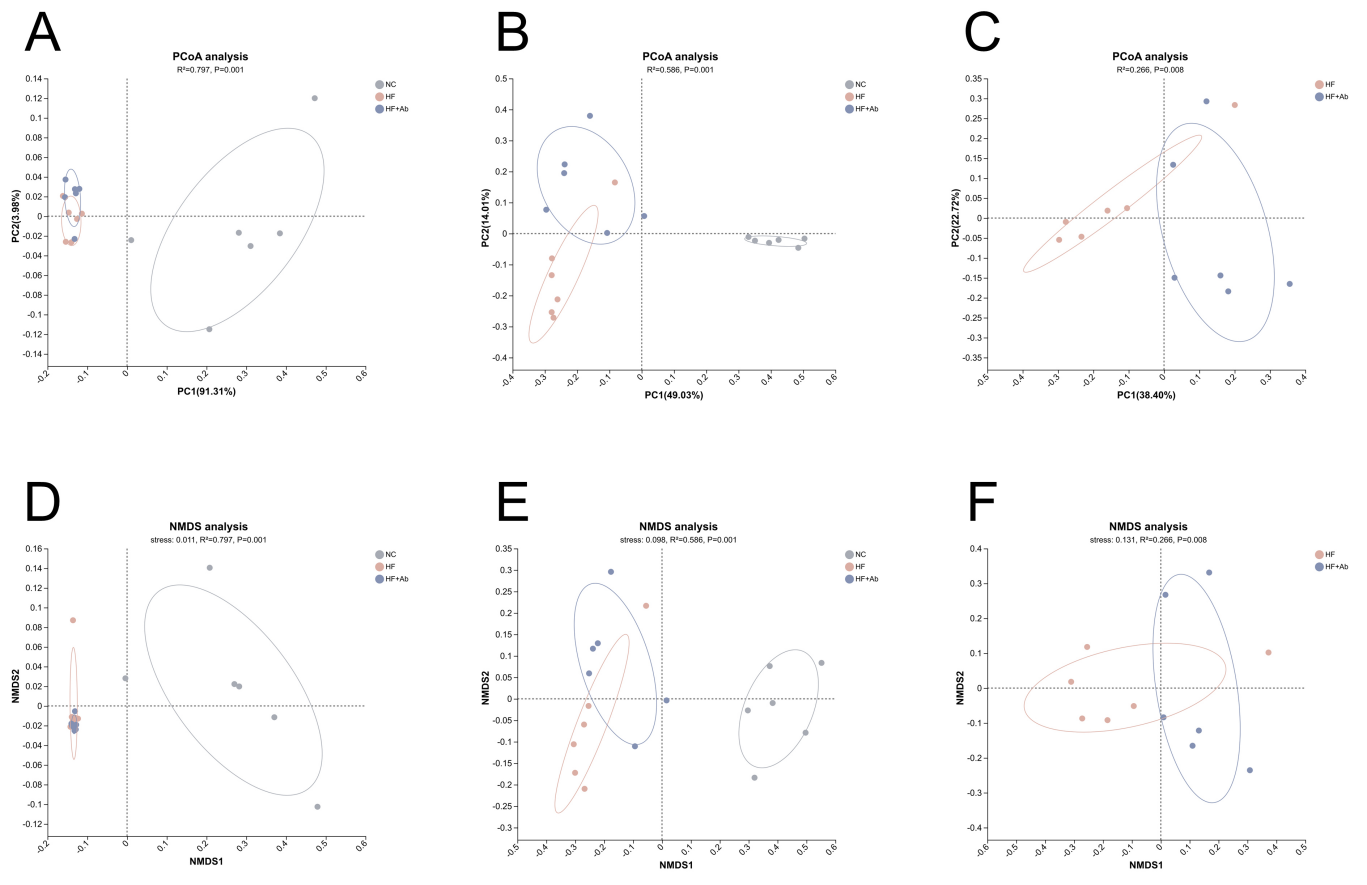
**Beta-diversity analysis of fecal metagenomic sequencing across groups**. PCoA is shown at the phylum level (A) and genus level (B), with an additional pairwise PCoA comparing HF vs. HF+Ab at the genus level (C). NMDS is shown at the phylum level (D) and genus level (E), with an additional pairwise NMDS comparing HF vs. HF+Ab at the genus level (F). Sample size: n = 6 per group. Statistics: Bray-Curtis dissimilarities were computed from relative abundance profiles; between-group differences were assessed by PERMANOVA. PCoA, principal coordinates analysis; NMDS, non-metric multidimensional scaling; PERMANOVA, permutational multivariate analysis of variance.

**Fig. 10.**
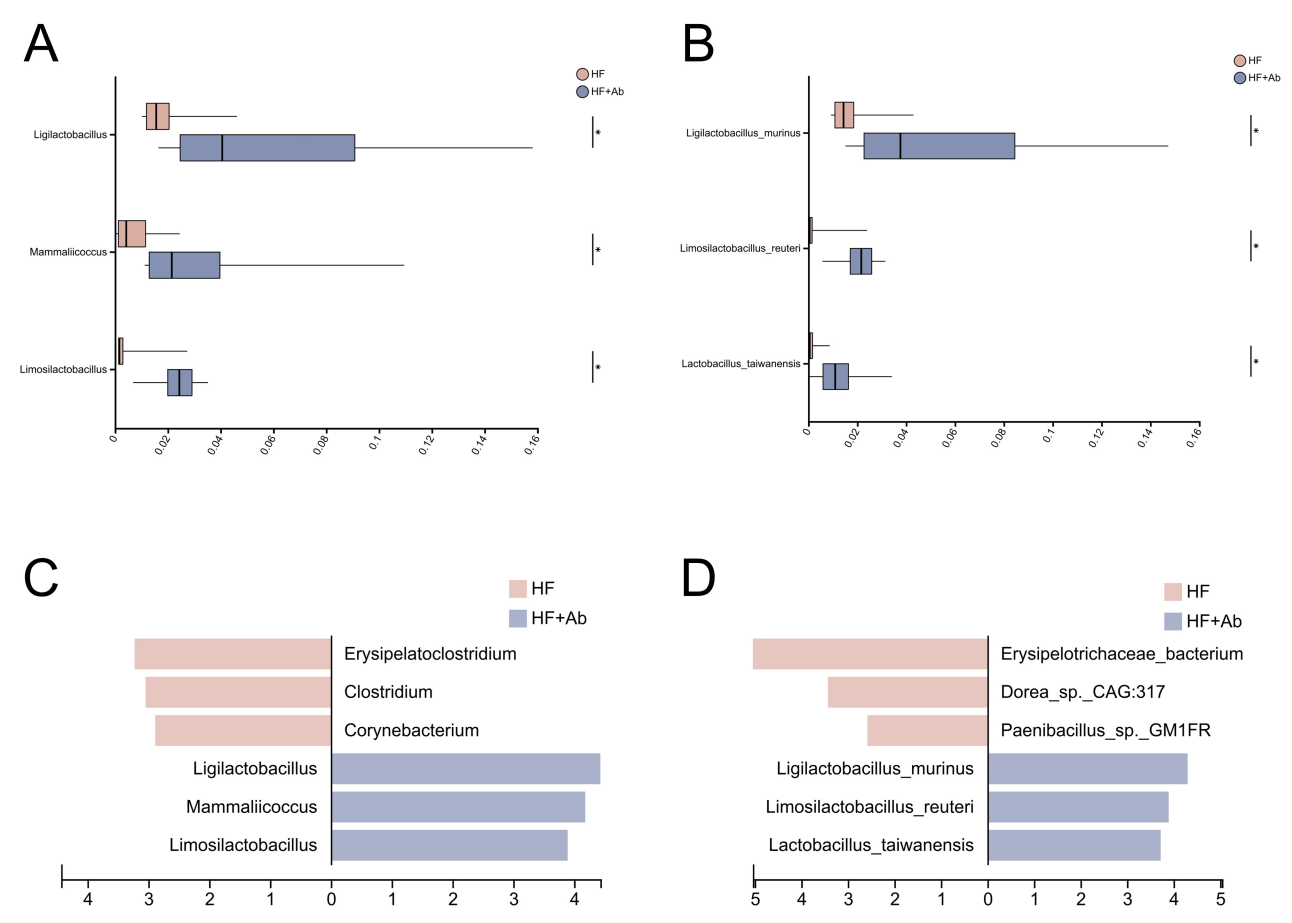
**Differential abundance analysis of the gut microbiota between the HF and HF+Ab groups**. Pairwise comparisons at the genus (A) and species (B) levels; LEfSe results (LDA scores) at the genus (C) and species (D) levels. Comparison among groups: *p < 0.05. Sample size: n = 6 per group. Statistics: Pairwise comparisons were performed using the Wilcoxon rank-sum test with FDR correction; LEfSe was conducted with α = 0.05 and an LDA score cutoff of >2.

For the antibody validation experiment (Fig. 1), six mice were randomly assigned to a 0.9% NaCl control group or the UC7-13D5 antibody-treated group (n = 3 per group). For the disease modeling and intervention experiments (Figs. 2,3,4,5,6,7,8,9,10), 36 mice were allocated to three groups (NCD, HFD, and HFD+Ab; n = 12 per group). For gut microbiome analyses (shotgun metagenomics), fecal samples were collected from all mice; however, due to cost considerations, a randomly selected subset of 6 mice per group was used for sequencing (n = 6 per group), as specified in the corresponding figure legends.

### 3.1 The Monoclonal Antibody UC7-13D5 Effectively Inhibits γδ T Cell Expression in ApoE^-⁣/-^ Mice

The effect of intraperitoneal injection of the monoclonal antibody UC7-13D5 was validated. Flow cytometry staining and statistical analysis demonstrated that the proportion of γδ T cells in both the peripheral blood (Fig. 1A) and intestinal tissue (Fig. 1C) of the antibody-treated group was significantly lower than that in the control group, with statistically significant differences (Fig. 1B,D). These findings indicate that UC7-13D5 effectively suppresses γδ T cell-associated signals detected by flow cytometry in mice, providing a foundation for subsequent studies.

### 3.2 Changes in Food Intake, Body Weight, and Lipid Levels in ApoE^-⁣/-^ Mice Under High-Fat Diet and UC7-13D5 Intervention

Food intake and body weight were monitored throughout the 12-week intervention, and longitudinal curves were plotted (Fig. 2B,C). During the intervention, the NC, HF, and HF+Ab groups showed broadly comparable overall levels of food intake as well as similar temporal trends, with no apparent group-specific intake pattern. Accordingly, the food intake curve is presented primarily to illustrate the overall trend over time. Body weight increased gradually over the intervention period in all groups. Overall, the trajectories of weight gain were similar among the three groups, and no evidence suggested a distinctly different longitudinal pattern of body weight change between groups.

Lipid analysis revealed that triglyceride levels (Fig. 3A) in the HF and HF+Ab groups were significantly higher than those in the NC group (*p* < 0.05). Additionally, triglyceride levels in the HF+Ab group were lower than those in the HF group, with statistically significant differences (*p* < 0.05). Similar results were observed for total cholesterol (Fig. 3B) and low-density lipoprotein cholesterol (Fig. 3C) (*p* < 0.05). However, no significant differences were observed in high-density lipoprotein cholesterol (Fig. 3D) levels among the three groups.

### 3.3 UC7-13D5 Effectively Suppresses High-Fat Diet-Induced Activation and Proliferation of γδ T Cells in ApoE^-⁣/-^ Mice

Flow cytometry analysis showed no significant differences in the proportion of CD3+ total T cells among the control group (NC), model group (HF), and model intervention group (HF+Ab) (Fig. 4A,E). In contrast, the proportion of γδ T cells (Fig. 4B,F) was significantly higher in the HF group compared to the NC group (*p* < 0.05). However, the HF+Ab group, treated with UC7-13D5, showed a significant reduction in γδ T cell proportions compared to the HF group (*p* < 0.05), reaching levels similar to the NC group. A similar trend was observed in the expression of IFN-γ+ γδ T cells (Fig. 4C,G) and IL-17A+ γδ T cells (Fig. 4D,H). The group differences in the proportion of IFN-γ+ γδ T cells were even more pronounced (*p* < 0.001).

### 3.4 UC7-13D5 Targeted Inhibition of γδ T Cells Improves Atherosclerosis in ApoE^-⁣/-^ Mice Under High-Fat Diet Conditions

Histological evaluation of the aorta and cross-sections of the aortic sinus was performed using Oil Red O staining, sliced Oil Red O staining, Masson staining, and HE staining. The HF group showed a significantly higher percentage of Oil Red O-positive staining on the aortic surface compared to the NC group (*p <* 0.001), while the HF+Ab group exhibited a significantly lower positive area compared to the HF group (*p* < 0.05) (Fig. 5A,E). In the aortic sinus, the HF group had a larger Oil Red O-positive area than the NC group (*p <* 0.001), and the HF+Ab group showed a further reduction compared to the HF group (*p* < 0.001) (Fig. 5B,F). The HF group displayed a higher positive area in Masson staining compared to the NC group (*p* < 0.001), while the HF+Ab group exhibited a lower positive area than the HF group (*p* < 0.05) (Fig. 5C,G). HE staining revealed a significantly higher necrotic core area percentage in the HF group compared to the NC group (*p <* 0.001), with the HF+Ab group showing a significant reduction compared to the HF group (*p* < 0.001) (Fig. 5D,H).

### 3.5 Targeted Inhibition of γδ T Cells Effectively Reduces Inflammatory Cytokine Expression in Peripheral Blood and Adipose Tissue

Analysis of inflammatory cytokines in peripheral blood revealed that TNF-α levels (Fig. 6A) were significantly elevated in the HF group compared to the NC group (*p* < 0.001). In the HF+Ab group, TNF-α levels were significantly lower than in the HF group (*p <* 0.05), although still higher than in the NC group. Similar trends were observed for IL-1β (Fig. 6B) and IL-6 (Fig. 6C) (*p* < 0.05). In addition, measurements of inguinal adipose tissue weight (Fig. 7A) and qPCR-based mRNA expression analyses of inflammatory cytokines showed that the HF group exhibited a significantly higher inguinal fat mass than the NC group, and that *TNF-α* (Fig. 7B) and *IL-6* (Fig. 7C) mRNA levels were also significantly elevated in the HF group compared with the NC group (*p* < 0.05). These levels were reduced in the HF+Ab group compared to the HF group (*p* < 0.05). These findings indicate that inhibition of γδ T cells alleviates inflammatory responses in adipose tissue.

### 3.6 Targeted Inhibition of γδ T Cells Modulates the Gut Microbiota Induced by a High-Fat Diet in ApoE^-⁣/-^ Mice

Alpha diversity analysis of fecal metagenomic sequencing revealed differences in microbial diversity across groups. Phylum level (Fig. 8A): The Shannon index of the NC group was significantly higher than that of the HF group (*p* < 0.01) and the HF+Ab group (*p* < 0.01). No significant differences were observed between the HF and HF+Ab groups. Genus level (Fig. 8B): The NC group showed a significant difference compared to the HF group (*p* < 0.01), but the difference between the NC group and the HF+Ab group was no longer statistically significant. No significant difference was observed between the HF and HF+Ab groups. Species level (Fig. 8C): The NC group exhibited a higher Shannon index than the HF group (*p* < 0.01) and the HF+Ab group (*p* < 0.05). Still, no significant difference was observed between the HF and HF+Ab groups.

Following the alpha-diversity analyses, beta diversity was evaluated using PCoA and NMDS based on Bray-Curtis dissimilarities.

In the PCoA ordinations, the NC group was clearly separated from both the HF and HF+Ab groups at the phylum level (Fig. 9A; R^2^ = 0.797, *p* = 0.001), whereas the HF and HF+Ab groups exhibited substantial overlap. At the genus level (Fig. 9B), the NC group remained distinct from the HF and HF+Ab groups, and separation between the HF and HF+Ab groups became evident (R^2^ = 0.586, *p* = 0.001). To focus on the diet-matched comparison, we performed a pairwise PCoA of the HF and HF+Ab groups at the genus level (Fig. 9C), confirming a significant difference in community structure (R^2^ = 0.266, *p* = 0.008).

To assess the robustness of the ordination patterns, NMDS was performed as a complementary approach and yielded results concordant with the PCoA. At the phylum level (Fig. 9D), the NC group separated from the HF and HF+Ab groups (stress = 0.011, R^2^ = 0.797, *p* = 0.001), while the HF and HF+Ab groups were not clearly distinguishable. At the genus level (Fig. 9E), NMDS revealed separation among all three groups (stress = 0.098, R^2^ = 0.586, *p* = 0.001). A pairwise NMDS analysis comparing HF and HF+Ab at the genus level (Fig. 9F) further supported a significant compositional difference between these two groups (stress = 0.131, R^2^ = 0.266, *p* = 0.008).

Following beta-diversity analyses of the fecal metagenomes, we observed significant differences in microbial community composition between the HF and HF+Ab groups at the genus level; therefore, differential taxonomic analyses were conducted at both the genus and species levels, including pairwise comparisons and LEfSe, to identify discriminative taxa.

At the genus level, pairwise differential abundance analysis (Fig. 10A) comparing the HF and HF+Ab groups identified *Ligilactobacillus*, *Mammaliicoccus*, and *Limosilactobacillus* as significantly enriched genera in the HF+Ab group (*p* < 0.05), and these genera ranked among the top three taxa showing the largest between-group differences in relative abundance. LEfSe analysis based on LDA scores (Fig. 10C) further ranked *Ligilactobacillus*, *Mammaliicoccus*, and *Limosilactobacillus* as the top three discriminative features for the HF+Ab group, consistent with the pairwise differential abundance results.

At the species level, pairwise differential abundance analysis (Fig. 10B) comparing the HF and HF+Ab groups showed that *Ligilactobacillus murinus*, *Limosilactobacillus reuteri*, and *Lactobacillus taiwanensis* were significantly enriched in the HF+Ab group and were among the top three differentially enriched species (*p* < 0.05). Consistently, LEfSe ranked these same species as the top three discriminative features for the HF+Ab group according to LDA scores (Fig. 10D).

Overall, genus- and species-level analyses consistently indicated that the HF+Ab group was characterized by a concordant set of enriched taxa, and the taxa identified by pairwise comparisons were corroborated by LEfSe-based LDA ranking.

## 4. Discussion

### 4.1 Effectiveness and Potential Limitations of UC7-13D5 in Suppressing γδ T Cell Expression in Mice

Flow cytometry analysis confirmed that the monoclonal antibody UC7-13D5 effectively inhibited γδ T cell expression in mice, consistent with previous studies [10, 11]. This provides a viable method for targeting γδ T cells and highlights the potential role of γδ T cells enriched in the intestinal epithelium in gut immunity. However, it is important to note the limitations of this antibody validation experiment. Although multiple research groups have used UC7-13D5 to intervene in γδ T cells and have reported significant biological effects, its mechanism of action should still be interpreted with caution and discussed in a pragmatic, evidence-based manner. A study suggests that it induces “functional exhaustion” of γδ T cells by blocking TCR signaling rather than directly depleting them [12]. This may affect the long-term efficacy and clarity regarding potential side effects. In addition, γδ T cells possess higher membrane cholesterol content and enriched lipid raft structures compared with conventional αβ T cells, which contributes to their heightened activation status [13]. Disruption of TCR signaling by UC7-13D5 may interfere with lipid raft organization, thereby attenuating downstream activation signals and inducing a functionally inactive or “invisible” state rather than true cellular depletion.

### 4.2 Effects of High-Fat Diet and UC7-13D5 on Food Intake, Body Weight, and Lipid Levels in ApoE^-⁣/-^ Mice

In the present study, food intake and body weight were monitored longitudinally during the 12-week intervention. Food intake showed broadly comparable trends across groups, and body weight increased over time in all groups, with no evidence of distinctly different longitudinal trajectories between groups. These observations suggest that the group differences observed in downstream outcomes between the HF and HF+Ab groups are unlikely to be explained by systematic differences in caloric intake or divergent body-weight trajectories during the intervention. Notably, while γδ T cells may have been implicated in metabolic regulation in other contexts, our current data do not allow us to draw firm conclusions regarding energy metabolism or fat accumulation based solely on food intake and body weight measurements, and this aspect warrants dedicated mechanistic investigation.

Lipid profiling showed that the high-fat diet markedly increased circulating TG, TC, and LDL-C. In the UC7-13D5-treated group, these lipid parameters exhibited a downward trend, suggesting that interference with γδ TCR signaling and the resultant functional attenuation of intestinal γδ T cells may indirectly influence systemic lipid metabolism. Given the established crosstalk between mucosal immunity and metabolic homeostasis, one plausible explanation is that dampening γδ T cell–driven intestinal inflammation could mitigate downstream inflammatory signaling, thereby improving metabolic derangements secondary to chronic low-grade inflammation [14, 15]. Importantly, UC7-13D5 selectively reduced LDL-C and triglycerides without significantly altering HDL-C. This pattern argues against a nonspecific reduction in intestinal lipid absorption as the sole mechanism, because generalized malabsorption would be expected to affect multiple lipid fractions rather than preferentially lowering apoB-containing lipoproteins. Instead, the selective reduction in LDL-C and TG is more consistent with modulation of pathways governing apoB-containing lipoprotein metabolism, including hepatic very low-density lipoprotein (VLDL) production and/or enhanced LDL clearance. In this regard, several gut–liver axis mechanisms could be implicated. For example, alterations in enteroendocrine signaling (e.g., glucagon-like peptide (GLP)-1) may influence hepatic lipid handling and VLDL–LDL flux [16]; similarly, changes in the proprotein convertase subtilisin/kexin type 9 (PCSK9)–LDLR axis could increase hepatic LDL uptake and lower circulating LDL-C without necessarily affecting HDL-C [17]. While these mechanistic links remain speculative in the absence of direct measurements, they provide a biologically plausible framework to reconcile the selective lipid pattern observed following UC7-13D5 intervention.

Our flow cytometry data showed reduced intestinal γδ T cell signals after antibody intervention, supporting the notion that UC7-13D5 effectively disrupts γδTCR-dependent activity within the gut compartment. Nevertheless, we acknowledge that the current data do not distinguish whether the observed metabolic improvement stems primarily from reduced intestinal inflammatory tone, altered hepatic lipid clearance, or combined effects across the gut–liver axis. Future studies incorporating targeted assessments of enteroendocrine mediators and hepatic lipid regulatory pathways, will be necessary to delineate the causal mechanisms linking γδ T cell perturbation to dyslipidemia under high-fat diet conditions.

### 4.3 Pathological Basis for the Improvement of Atherosclerotic Lesions in ApoE^-⁣/-^ Mice by Targeted Inhibition of γδ T Cells 

Oil Red O staining revealed a significant increase in aortic lipid deposition in the HF group, while lipid deposition was reduced in the HF+Ab group after UC7-13D5 intervention. This indicates that γδ T cell inhibition is associated with reduced atherosclerotic lesions. Further analysis using Masson and HE staining showed that collagen fibrosis and necrotic cores were markedly increased in the HF group but relatively reduced in the HF+Ab group. These findings suggest that targeted inhibition of γδ T cells may reduce vascular inflammation by lowering the expression of pro-inflammatory cytokines IFN-γ and IL-17A [18, 19]. The results of this study support immune regulation mediated by γδ T cells as a potential therapeutic approach for AS [20, 21, 22].

Mechanistically, the reduction in lesion burden observed with UC7-13D5 is consistent with the broader concept that inflammation is a key driver of atherosclerotic plaque progression and vulnerability [23]. In particular, inflammasome signaling and the IL-1 pathway have been repeatedly implicated in atherogenesis: cholesterol crystals can activate the NLR family pyrin domain containing 3 (NLRP3) inflammasome, promoting IL-1β maturation and downstream inflammatory amplification within lesions [24]. Beyond cytokine production, pyroptotic executioners such as gasdermin D have also been linked to plaque inflammation and phenotype transitions, supporting a role for inflammasome-gasdermin signaling in shaping inflammatory and potentially more vulnerable plaques [25]. Our observation that UC7-13D5 treatment decreased IL-1β and IL-6 in peripheral blood and adipose tissue is therefore compatible with attenuation of an IL-1–centric inflammatory network, which could contribute to reduced atherosclerotic lesion burden. At the clinical level, residual inflammatory risk remains a major determinant of adverse cardiovascular outcomes even among patients receiving contemporary lipid-lowering therapy, underscoring the relevance of targeting inflammatory pathways in addition to cholesterol management [26].

### 4.4 Effects of a High-Fat Diet on Gut Microbiota in ApoE^-⁣/-^ Mice and the Potential Benefits of Targeting γδ T Cells 

The gut microbiota findings in our study are particularly relevant in light of accumulating evidence that intestinal microbes can modulate atherosclerosis and cardiometabolic risk through defined host–microbe metabolic pathways. A seminal study demonstrated that gut microbiota–dependent metabolism of dietary phosphatidylcholine generates trimethylamine (TMA), which is subsequently converted by host hepatic enzymes into trimethylamine N-oxide (TMAO), a metabolite that promotes macrophage foam cell formation and accelerates atherosclerotic lesion development, suppression of intestinal microbiota attenuated choline-driven atherosclerosis in susceptible mouse models [27]. Subsequent work has further synthesized mechanistic and clinical evidence supporting the contributory role of the gut microbiota–TMA/TMAO axis in cardiovascular disease and atherosclerosis risk [28].

In our study, compared to the normal diet group, a high-fat diet significantly reduced gut microbial diversity in *ApoE*^-⁣/-^ mice. Within this context, our observation that UC7-13D5 treatment is associated with altered gut microbial community structure suggests a potential immunometabolic link between intestinal γδ T cell signaling and the microbial ecosystem. Notably, UC7-13D5 treatment was accompanied by an increased relative abundance of commonly regarded as beneficial taxa, particularly *Ligilactobacillus murinus* and *Limosilactobacillus reuteri*. This compositional shift may be biologically relevant, as *Ligilactobacillus murinus* has been reported to attenuate atherosclerosis by suppressing macrophage pyroptosis through a butyrate-GPR109A-GSDMD axis [29]. In parallel, *Limosilactobacillus reuteri* is a widely used probiotic with well-described, context-dependent immune-regulatory effects, providing a plausible link between its enrichment and the immunomodulatory phenotype observed after UC7-13D5 intervention [30]. Collectively, the observed compositional improvements in the gut microbiota in the context of a high-fat diet were linked to targeted inhibition of γδ T-cell function [31]. But these observations are correlative and do not establish causality.

### 4.5 Limitations

We also acknowledge several limitations in our study. First, our analyses were conducted at specific time points and did not capture long-term dynamic changes in the microbiota. Second, we did not establish a direct causal relationship between UC7-13D5-mediated γδTCR blockade and microbial alterations. The present findings are primarily based on associative observations, and no direct mechanistic experiments were performed to demonstrate that γδ T cells actively modulate gut microbiota composition to drive atherosclerotic progression. Therefore, the proposed γδ T cell-gut microbiota-atherosclerosis axis should be interpreted with caution and regarded as a hypothesis-generating framework rather than a definitive causal pathway. Future studies should incorporate longitudinal sampling and causal validation strategies, such as fecal microbiota transplantation, targeted metabolite profiling (e.g., TMAO, bile acids, and short-chain fatty acids), and functional/strain-level metagenomic analyses to clarify whether microbiota changes mediate, or merely accompany, the vascular and metabolic phenotypes observed following UC7-13D5 intervention.

## 5. Conclusion

UC7-13D5–mediated inhibition of γδ T cells attenuated high-fat diet–associated inflammatory cytokine expression and was associated with improved lipid metabolism, shifts in gut microbial community structure, and reduced atherosclerotic lesion burden in mice. These findings implicate γδ T cells in cardiometabolic inflammation and warrant further evaluation of γδ T-cell–targeted strategies for atherosclerosis.

## Availability of Data and Materials

The datasets used and analysed during the current study are available from the corresponding author on reasonable request.
